# Travel Medicine: Tales Behind the Science

**DOI:** 10.3201/eid1403.071595

**Published:** 2008-04

**Authors:** I. Dale Carroll

**Affiliations:** * The Travel Doctor, Granville, Michigan, USA

**Keywords:** Vaccine, travel, migration, history, book review

This book is a compilation of 40 essays written by many of the most recognized names in the field of travel medicine. It is divided into 9 sections with such topics as the history of travel medicine, vaccines, travel medicine research, pilgrimages, and even space travel. Photographs, tables, and charts enhance the reader’s interest, especially when one spots a familiar person or place.

The styles range from the didactic, to short story to poetry, and the mood ranges from the humor of Jay Keystone’s “Ten Commandments” to the stark reality of Marc Shaw’s “Amazonas Adventure.” One cannot help but chuckle at Charles Ericsson’s description of diarrhea research or laugh outright at Steve Toovey’s “Woman Atop the Crocodile,” and Nancy Piper Jenks’ account of undocumented migrants may bring the reader to tears.

This is not a formal textbook of travel medicine, but much can be learned from it. Although not a history text, the book is replete with fascinating accounts of medical history. One learns such things as the origin of the word “quarantine,” the complexities of preparing a certification examination, and the sheer terror of being on the front lines of an epidemic of severe acute respiratory syndrome. In short, the volume explains why things are the way they are in travel medicine and why this new discipline has, of necessity, become a separate specialty.

The essays need not be read in the order presented, but surprisingly, some of the topics that seem least interesting turn out to be the most fascinating. Much of the book reads like a medical detective story; other parts read like a medical journal but the writing is more compelling.

If I had to produce a criticism for the book, it would be simply that the publisher has picked a size of print that is almost too small for my presbyopic eyes. Overall, the book is a fascinating read, and one can only hope that future editions will be forthcoming.

